# Skin Toxicity of Selected Hair Cosmetic Ingredients: A Review Focusing on Hairdressers

**DOI:** 10.3390/ijerph19137588

**Published:** 2022-06-21

**Authors:** Cara Symanzik, Patricia Weinert, Željka Babić, Sarah Hallmann, Martin Stibius Havmose, Jeanne Duus Johansen, Sanja Kezic, Marija Macan, Jelena Macan, Julia Strahwald, Rajka Turk, Henk F. van der Molen, Swen Malte John, Wolfgang Uter

**Affiliations:** 1 Institute for Interdisciplinary Dermatological Prevention and Rehabilitation (iDerm) and Department of Dermatology, Environmental Medicine and Health Theory, Osnabrück University, 49076 Osnabrueck, Germany; patricia.weinert@uni-osnabrueck.de (P.W.); sjohn@uni-osnabrueck.de (S.M.J.); 2 Institute for Medical Research and Occupational Health, HR 10001 Zagreb, Croatia; zbabic@imi.hr (Ž.B.); mmacan@imi.hr (M.M.); jmacan@imi.hr (J.M.); rturk@imi.hr (R.T.); 3 Department of Medical Informatics, Biometry and Epidemiology, University of Erlangen, 91054 Erlangen, Germany; sarah.hallmann@fau.de (S.H.); julia.strahwald@outlook.de (J.S.); wolfgang.uter@fau.de (W.U.); 4 National Allergy Research Centre, Department of Skin and Allergy, University of Copenhagen, Gentofte Hospital, 2900 Copenhagen, Denmark; martin.stibius.havmose@regionh.dk (M.S.H.); jeanne.duus.johansen@regionh.dk (J.D.J.); 5 Amsterdam UMC, Department of Public and Occupational Health, Coronel Institute of Occupational Health, Amsterdam Public Health Research Institute, University of Amsterdam, 1105 AZ Amsterdam, The Netherlands; s.kezic@amc.uva.nl (S.K.); h.f.vandermolen@amsterdamumc.nl (H.F.v.d.M.)

**Keywords:** cysteamine hydrochloride, cocamide diethanolamine, cocamidopropyl betaine, cosmetics, hairdresser, hairdressing, hand eczema, polyvinylpyrrolidone, polyvinylpyrrolidone copolymers, sodium laureth sulfate

## Abstract

The safety assessment of cosmetics considers the exposure of a ‘common consumer’, not the occupational exposure of hairdressers. This review aims to compile and appraise evidence regarding the skin toxicity of cysteamine hydrochloride (cysteamine HCl; CAS no. 156-57-0), polyvinylpyrrolidone (PVP; CAS no. 9003-39-8), PVP copolymers (CAS no. 28211-18-9), sodium laureth sulfate (SLES; CAS no. 9004-82-4), cocamide diethanolamine (cocamide DEA; CAS no. 68603-42-9), and cocamidopropyl betaine (CAPB; CAS no. 61789-40-0). A total of 298 articles were identified, of which 70 were included. Meta-analysis revealed that hairdressers have a 1.7-fold increased risk of developing a contact allergy to CAPB compared to controls who are not hairdressers. Hairdressers might have a higher risk of acquiring quantum sensitization against cysteamine HCl compared to a consumer because of their job responsibilities. Regarding cocamide DEA, the irritant potential of this surfactant should not be overlooked. Original articles for PVP, PVP copolymers, and SLES are lacking. This systematic review indicates that the current standards do not effectively address the occupational risks associated with hairdressers’ usage of hair cosmetics. The considerable irritant and/or allergenic potential of substances used in hair cosmetics should prompt a reassessment of current risk assessment practices.

## 1. Introduction

Hairdressers are a high-risk group for acquiring occupational skin diseases (OSD) as a result of work-related damage to the skin. Hand dermatitis (hand eczema, HE) is therefore the most frequent OSD [1,2]. A current systematic review and meta-analysis of published literature from 2000–2021 has shown that there is a pooled lifetime prevalence of 38.2% and a 1-year prevalence of 20.3% of HE in hairdressers [3]. Wet work and skin contact with irritants and allergens are the most crucial factors in the development of HE in this occupational area [4,5,6]. Numerous main ingredients in various hair cosmetics are strong allergens [7]. The deterioration of the epidermal barrier function brought about by the prevailing work routines of hairdressers, combined with the initiation of a proinflammatory milieu, increases the risk of developing occupational contact dermatitis. Because allergens penetrate the compromised skin barrier more swiftly, initial irritant HE can easily progress to the development of allergic HE [8]. This should be regarded as extremely problematic because OSD not only cause individual suffering resulting from illness, but also pose a macrosocial problem, since OSD may lead to elevated medical treatment costs for the social insurance system, and may have social implications such as the need for a change of occupation or, in the worst case, early retirement [9,10,11]. The considerable public health impact resulting from contact allergy (sensitization) and allergic contact dermatitis has been detailed in a recent paper by Uter et al. [12].

In contrast to most customers using cosmetics only for a few minutes each day, hairdressers may be exposed to them for up to eight hours per day, five or six days per week, throughout their careers. Since the Scientific Committee on Consumer Safety (SCCS) does not have a mandate to assess risk specifically of occupational exposures, but of general consumer exposures, most scientific SCCS opinions do not address the significant excess in exposure to hazardous substances of a hairdresser [13]. Similarly, the European Cosmetics Regulation, which was adopted in 2009, is principally intended to safeguard consumers, with only a few provisions for professional users, and is therefore unfit to adequately address risks associated with the occupational use of cosmetic substances for hairdressers [14].

Within the framework of the project, “Promoting the autonomous implementation of the European framework agreement on occupational health and safety in the hairdressing sector”, a series of systematic reviews has been performed, synthesizing evidence on several key hazardous substances [3,15,16,17,18,19]. As further important, but less well-investigated ingredients of hair cosmetics, cysteamine hydrochloride (cysteamine HCl; CAS no. 156-57-0), polyvinylpyrrolidone (PVP; CAS no. 9003-39-8), polyvinylpyrrolidone (PVP) copolymers (CAS no. 28211-18-9), sodium laureth sulfate (SLES; CAS no. 9004-82-4), cocamide diethanolamine (cocamide DEA; CAS no. 68603-42-9), and cocamidopropyl betaine (CAPB; CAS no. 61789-40-0) were identified by a so-called Delphi Process (i.e., a tried and tested methodology used for the elicitation of opinions of experts). Thus, the present review aims to compile and appraise clinical evidence regarding the skin toxicity of cysteamine HCl, PVP, PVP copolymers, SLES, cocamide DEA, and CAPB contained in hair cosmetics.

## 2. Materials and Methods

### 2.1. Registration and Protocol

The present review was registered in the International Prospective Register of Systematic Reviews (PROSPERO; registration number CRD42021238118) [20]. The protocol is published elsewhere [15]. No changes were made to the information provided at registration or in the protocol.

### 2.2. Eligibility Criteria

Eligibility criteria for the studies to be included in the present review are reported following the PECOS scheme, adapted from the CRD’s (Centre for Reviews and Dissemination) guidance for undertaking reviews in health care [21] (Table 1).

### 2.3. Information Sources

The online database Pubmed/Medline was used to conduct systematic searches. We undertook a narrative synthesis of the data rather than a meta-analysis, since we expected considerable variation in methods and outcomes, except when quantitative summary statistics were possible.

### 2.4. Search Strategy

Searches were conducted in November 2021. In addition, we hand-searched the bibliographies of all the papers that met the inclusion criteria and were found through an electronic database search (backward snowballing). We also used forward snowballing to check all references quoting any of these articles, using six relevant references [6,22,23,24,25,26]. This citation analysis was conducted using the Pubmed/Medline database. Only English search phrases were utilized. We looked for the title, abstract, and important terms in general. Only peer-reviewed articles from 1991 onwards were considered. For PVP copolymers, we included PVP copolymers listed in the International Nomenclature Cosmetic Ingredients (INCI) inventory [27]. The full search strings for all six substances addressed in this review can be found in Appendix A.

### 2.5. Selection Process

The search results from Medline were exported in an appropriate format and imported into separate Zotero libraries for each search query (see also Table 1), with the number of references of each ex-/import set documented. Bibliographical duplicates were determined and excluded in the Zotero library [15]. Non-inclusion reasons were noted and summarized at the conclusion of the process for use in the PRISMA-P flowchart [28].

After the first sets of references, one for each substance, had been preserved, the final sets of references, appropriate for full text screening by two reviewers, were imported into a Zotero cloud-based reference database. Two reviewers separately analyzed and extracted full text articles (C.S. and P.W.), with a third senior reviewer reconciling different results between the two original reviewers (W.U. or S.M.J.). All judgments and reasons for the exclusion of studies were documented, including information on the first reviewers’ individual assessments as well as the final decision. A collection of full text articles to be included in the systematic review was thereby defined at the end of this procedure.

### 2.6. Data Collection Process and Data Items

Using a standardized, pre-piloted publication record form (PRF), two reviewers independently retrieved data from publications that met the inclusion criteria (C.S. and P.W.). In situations with conflicting data, a third senior reviewer joined the decision discussion and made final decisions (W.U. or S.M.J.). The finished PRF was saved and is included as additional material (Appendix B). For original articles, the following basic data were extracted: publication ID, year of study execution, country of origin, study design, methods, study setting and population involved, information on the basic characteristics of participants (i.e., age, sex), number of participants, and clinical outcome(s). Data on skin toxicity were sought for the outcome of skin sensitization/contact allergy in humans (e.g., numbers tested, numbers positive, test methods). For case reports and case series, the following data were summarized: publication ID, year of study execution, country of origin, information on the basic characteristics of participants (i.e., age, sex, occupation, working years), and patient-specific products tested along with the results obtained with these.

### 2.7. Effect Measures

The primary effect measure was the prevalence of sensitization diagnosed by patch testing clinical samples (patients with suspected allergic contact dermatitis potentially sensitized to the tested substances, e.g., by exposure to hair cosmetics). The clinical samples were further stratified, where possible, for hairdressers vs. other patients. If stratification was possible within one study, risk quotients were derived in terms of relative risk (RR), formally estimated by a prevalence ratio (PR) [15], that is, by dividing the prevalence in hairdressers by the prevalence in the respective control group. A pooled estimate is provided if homogeneity of single estimates permits.

### 2.8. Synthesis Methods

It was expected that approaches and outcomes would be mostly heterogeneous. Hence, instead of quantitatively pooling results in a meta-analysis, a narrative synthesis in accordance with the CRD’s recommendations was performed. [21]. The essential characteristics of the included research, as well as their conclusions, are presented in summary tables. Forest plots with an estimate of heterogeneity (I^2^) are used to give graphical summaries where indicated and possible.

## 3. Results

### 3.1. Study Selection and Study Characteristics

**Figure 1:**
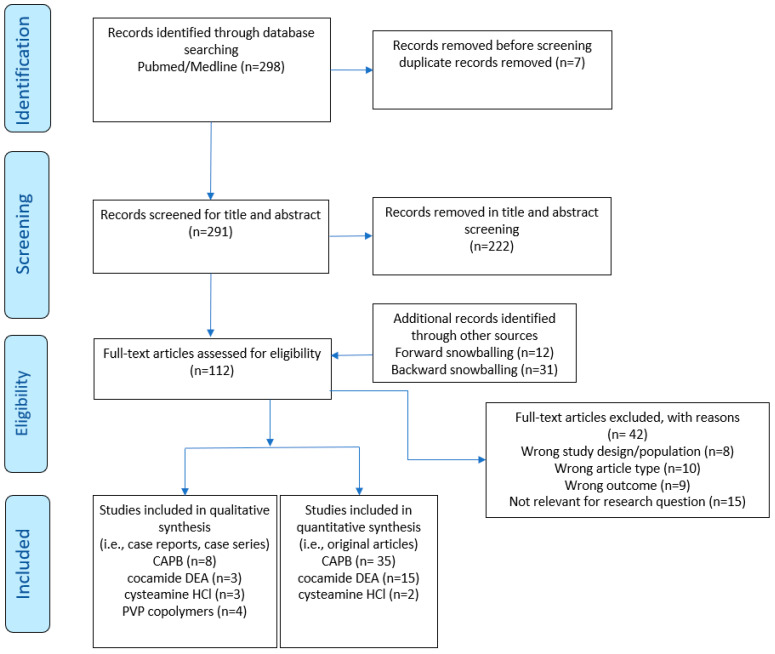
Preferred Reporting Items for Systematic Reviews and Meta-Analyses (PRISMA) 2020, flow diagram of literature search according to Page et al. [38].

Figure 1 displays a flow diagram of the study selection. Initial searches overall provided 298 study records. Following title and abstract screening and the removal of bibliographic duplicates, 112 records were left to be screened on the full-text level, including another 43 references identified by manually searching the references (i.e., backward and forward snowballing). A number of studies which appeared to meet the inclusion criteria at first screening had to be omitted upon full-text scrutiny since they focused on a population that was inappropriate for this review. Indicative reasons for exclusion were a wrong outcome [29,30], a wrong study population [31,32], a wrong article type [33,34], or not being relevant to the research question [35,36,37]. Case reports and case series were compiled and extracted as supplemental information in case they were eligible in terms of the inclusion criteria. Overall, we arrived at a final number of 70 papers.

Characteristics of the included studies were recorded using PRFs (Appendix B). A total of 35 original articles on CAPB [6,25,26,39,40,41,42,43,44,45,46,47,48,49,50,51,52,53,54,55,56,57,58,59,60,61,62,63,64,65,66,67,68], 15 on cocamide DEA [37,40,41,42,43,57,60,63,65,66,67,69,70,71,72], and 2 on cysteamine HCl [56,73] were included; all of them being patch test studies. Regarding case reports and case series, 8 publications on CAPB [74,75,76,77,78,79,80,81], 3 on cocamide DEA [82,83,84], 3 on cysteamine HCl [85,86,87], and 4 on PVP copolymers [88,89,90,91] were included.

### 3.2. Results of Individual Studies

#### 3.2.1. Cysteamine Hydrochloride

Original articles regarding patch testing for cysteamine HCl are summarized in Table A1. Ito et al. analyzed patch test data from 2012 and 2014 in a multi-institutional joint study in Japan, with the aim of investigating which ingredients caused allergic contact dermatitis related to hair dye and perming solutions in Japan, to assess whether PPD is suitable for screening for hair dye allergy, and to propose allergens for a Japanese hairdresser series [73]. A total of 26 of 192 (13.5%) patients were found to be allergic to cysteamine HCl; among these 8/26 were hairdressers and 18/166 were non-occupationally exposed (PR: 2.84, 95%CI: 1.28–6.0) [73]. Schwensen et al. examined data from 2002 to 2011 of the Danish Contact Dermatitis Group, with the aim of identifying sensitization to the most common allergens associated with hairdressing [56]. Of the hairdressers tested with cysteamine HCl, 1 of 12 (of a total of 399) was positive (8.3%, 95% CI: 0–24%) [56].

Case reports and case series on sensitization against cysteamine HCl are summarized in Table A2. In 2004, Isaakson and van der Walle reported on a 53-year-old female hairdresser in Sweden with positive patch test results against cysteamine HCl (patient-specific product tested: perm solution with the subsequent testing of cysteamine HCl, 1.0% pet., as an individual substance) [85]. Landers, Law, and Storrs presented a case of a 38-year-old female hairdresser in 2002 in the USA who was tested positive for cysteamine HCl (patient-specific product tested: perm solution with the subsequent testing of cysteamine HCl, 0.5% pet. and 1.0% pet., as an individual substance) [86]. From 2012 to 2017, Nishioka, Koizumi, and Takita reported on seven cases of hairdressers (three males and four females; age range: 22 to 73 years) in Japan who were patch tested positive for cysteamine HCl [87]. None of the mentioned case reports and case series reported on the working years of the hairdressers [85,86,87].

#### 3.2.2. Polyvinylpyrrolidone

Neither original articles nor case series or case reports regarding pertinent skin toxicity of PVP were found. Thus, no data on PVP can be included.

#### 3.2.3. Polyvinylpyrrolidone Copolymers

No original articles, but only case reports were found regarding the skin toxicity of PVP copolymers, which are summarized in Table A3. In 2021, Buonomo and Warshaw reported on a case of a 25-year-old female in the USA with a positive patch test result against PVP copolymers (patient-specific product tested: moisturizer with the subsequent testing of PVP/eicosene copolymer (Ashland Inc., Wilmington, Delaware), 10% pet., as an individual substance) [88]. Pastor et al. presented a case of a 20-year-old woman in Spain showing a positive patch test reaction against PVP copolymers (patient-specific product tested: lipstick with the subsequent testing of PVP/hexadecene copolymer, 5% pet., provided by the manufacturer of the lipstick as an individual substance) in 2008 [89]. In 2006, Quartier et al. presented a 28-year-old woman in Belgium with a positive patch test reaction against PVP copolymers (patient-specific product tested: lipstick with the subsequent testing of PVP/hexadecene copolymer, 10% pet., 5% pet., and 1% pet., as well as PVP/eicosene copolymer, 10% pet., 5% pet., and 1% pet., provided by the manufacturer of the lipstick as individual substances) [90]. In 1998, Scheman and Cummins reported the case of a 53-year-old woman in the USA showing a positive patch test reaction to PVP copolymers (patient-specific product tested: skin care products with the subsequent testing of PVP/hexadecene copolymer, 5% pet., as an individual substance) [91]. In all the aforementioned case reports, the occupation as well as the working years of the tested patients are not specified [88,89,90,91].

#### 3.2.4. Sodium Laureth Sulfate

Neither original articles nor case series or case reports regarding the relevant skin toxicity of SLES were found. Thus, no data on SLES can be included.

#### 3.2.5. Cocamide Diethanolamine

All the information on original articles regarding patch testing for cocamide DEA are summarized in Table A4. None of the original articles regarding cocamide DEA provided data specifically for hairdressers, except for the paper by Mertens, Gilissen, and Goossens [71], which is why in the course of this subchapter mostly the positive reactions of patients other than hairdressers are described (Table 2). In the study by Mertens, Gilissen, and Goossens, 6 of 18 (33.3%) cocamide DEA-sensitized individuals worked as hairdressers; shampoos and hand cleansers were identified as culprit exposures [71]. Grey et al. conducted a double-blind randomized controlled study recruiting previously patch-tested patients who had been “surfactant-positive” and assessed co-reactivity to novel surfactant allergens, from 2015 to 2016 in the USA. They found that 4 of 47 (8.5%) study participants showed a positive patch test result regarding cocamide DEA, although not coupled with other surfactants [70].

Case reports and case series on sensitization regarding cocamide DEA are summarized in Table A5. In 1998, Fowler presented a case series of three people (one woman and two men, aged 40, 47, and 28 years) with an allergy to cocamide DEA in the USA (patient-specific product tested: personal care products with the subsequent testing of cocamide DEA, 0.5% pet., as an individual substance) [84]. In 2005, Dejobert et al. described a case of a 27-year-old woman with a cocamide DEA allergy in France (patient-specific product tested: shampoo with the subsequent testing of cocamide DEA, 0.5% pet., as an individual substance) [83]. In 2015, Badaoui et al. reported on a series of six cases (four females and two males with a mean age of 51.6 years) of allergies to cocamide DEA (patient-specific product tested: antifungal cream and disinfection spray with the subsequent testing of cocamide DEA, 0.5% pet., as an individual substance) [82]. None of the aforementioned case reports and case series provided data specifically for hairdressers; the occupations (except for one manufacturer and one mechanic) and working years remain unclear.

#### 3.2.6. Cocamidopropyl Betaine

**Figure 2:**
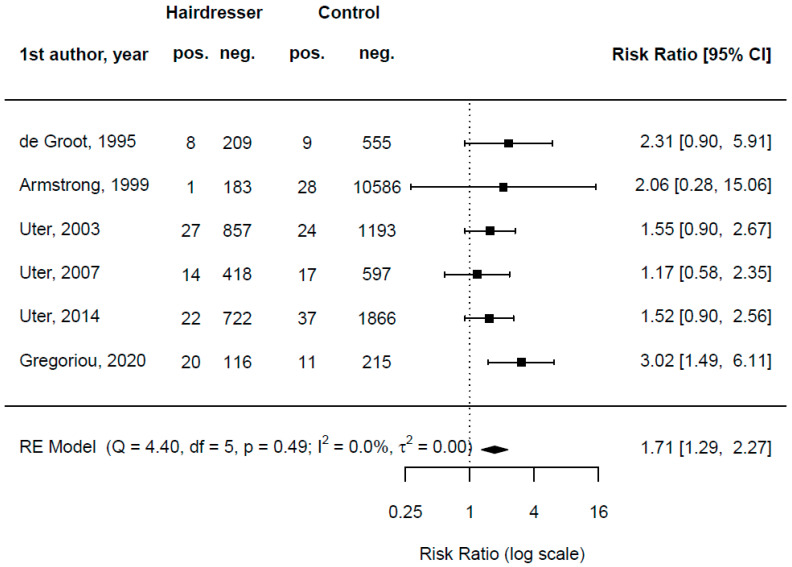
Forest plot quantifying the risk of cocamidopropyl betaine (CAPB) contact allergy diagnosed by patch testing associated with being a hairdresser vs. other (non-specified) occupation/exposure. De Groot et al. restricted “positive” reactions to “clinically relevant positive” reactions [26]; Armstrong et al. compared hairdressers to consecutively patch-tested patients [25]; and the three Uter et al. studies compared female hairdressers to female patients with suspected contact dermatitis to hair cosmetics—mostly dyes, bleaches, and waving products [22,23,24].

A summary of the original articles regarding CAPB can be found in Table A6. Six original articles provided data on sensitization against CAPB in hairdressers as well as others (controls). De Groot, van der Walle, and Weyland looked at patch test data from 1991–1994 from the Netherlands and reported that 8 of 217 (3.7%) hairdressers as well as 9 of 564 (1.6%) other patients provided positive patch test reactions for CAPB, with shampoos being identified as culprit exposures [26]. Gregoriou et al. retrospectively reviewed the medical records of patients with suspected allergic contact dermatitis to hair dyes from 2010 to 2019 from Greece and found that 20 of 136 (14.7%) hairdressers as well as 11 of 226 (4.9%) other patients showed positive CAPB patch test results, with hair dyes identified as the main culprit exposure [45]. Armstrong et al. reported data from 1991 to 1998 in the UK (St. John’s, London) and found that 1 of 184 (0.54%) hairdressers had a positive patch test reaction regarding CAPB, whereas 28 of 10,614 (0.26%) other patients had a positive patch test reaction regarding CAPB [25]. Of note, in the initial study period, a Tegobetaine product had been used, which, according to the authors, contained more by-products than the later used test substance; this supposedly relates to a much higher frequency of positive CAPB reactions in the former period (24/6042 vs. 5/4756). In three subsequent study periods, Uter et al. compared sensitization frequencies between female hairdressers and female clients/self-users, to a hair cosmetic series including CAPB 1% aq. in the Information Network of Departments of Dermatology (IVDK) [22,23,24]; the results are also illustrated in Figure 2 in terms of a forest plot. While in none of the single studies is the moderate increase in risk associated with hairdressing significant, except for Gregoriou et al. [45], the pooled estimate indicates a significantly increased risk. The pooled risk ratio is 1.71 [1.29, 2.27]. Thus, hairdressers seem to have a 1.7-fold increased risk of developing a contact allergy to CAPB compared to controls who are not hairdressers.

**Figure 3:**
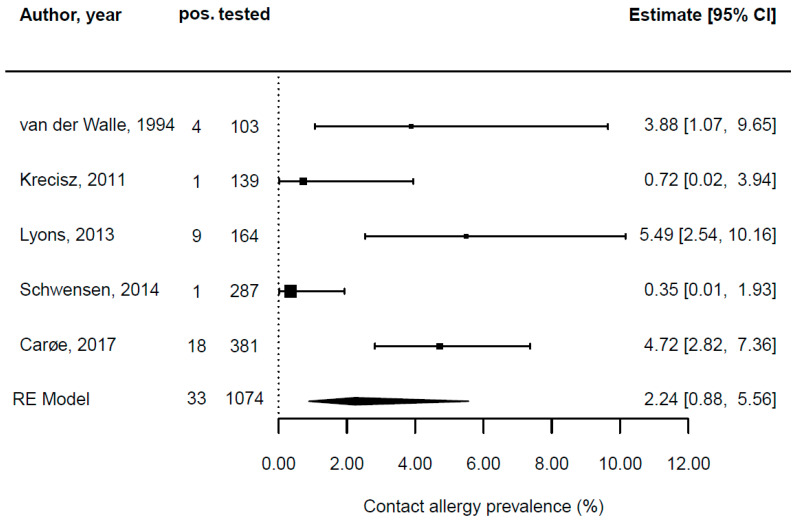
Forest plot depicting the prevalence of cocamidopropyl betaine (CAPB) contact allergy diagnosed by patch testing in hairdressers [6,48,50,56,62].

Five original articles only provided data on hairdressers and not on other patients. With a study period from 1989 to 1992 in the Netherlands, van der Walle and Brunsveld reported on 4 of 103 (3.9%) hairdressers giving a positive patch test reaction regarding CAPB, with hairdressing products being identified as the main source of exposure [62]. Lyons et al. looked at patch test data from 1993 to 2010 from Australia and concluded that of 164 hairdressers, 9 (5.5%) presented with a positive patch test result for CAPB, with hairdressing products being identified as the culprit exposure [50]. Schwensen et al. analyzed patch test data from the Danish Contact Dermatitis Group from 2002 to 2011 in Denmark and reported that 1 of 287 (0.3%) hairdressers showed a positive patch test reaction regarding CAPB, with hairdressing products being identified as the main exposure [56]. In 2011, Krecisz, Kiec-Swierczynska, and Chomiczewska found positive patch test results for CAPB in 1 of 139 (0.7%) hairdressing apprentices in Poland, again with hair cosmetics being the culprit exposure [48]. Carøe, Ebbehøj, and Agner conducted a descriptive, register-based survey of patch test data from 2006 to 2011 from Denmark and found that 18 of 381 (4.7%) of the patch-tested hairdressers had shown a positive result for CAPB, with surfactants being the principal source of exposure [6]. Figure 3 depicts the pooled CAPB contact allergy prevalence in hairdressers, which is 2.2%.

**Figure 4:**
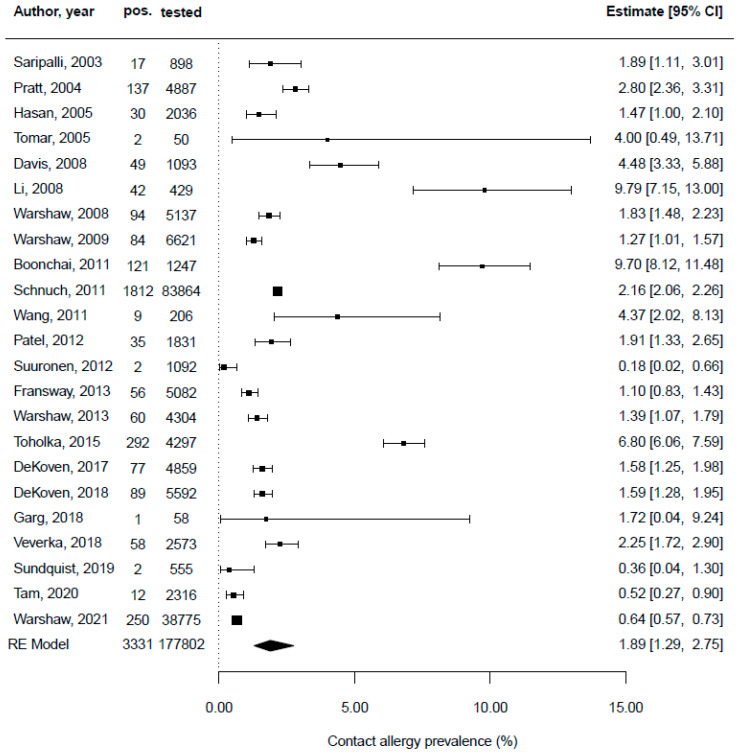
Forest plot depicting the prevalence of cocamidopropyl betaine (CAPB) contact allergy diagnosed by patch testing in patients other than hairdressers or with non-specified occupation/exposure [39,40,41,42,43,44,46,49,51,52,54,55,57,58,59,60,61,63,64,65,66,67,68].

Twenty-four original articles provided data on other patients than hairdressers. Patel and Belsito described patch test data from 1995 to 2010 from the USA and found that 35 of 1831 (1.9%) patients reacted positive against CAPB [51]. Hasan et al. examined patch test reactions to cosmetic allergens from 1995 to 1997 and 2000 to 2002 in Finland and reported on 30 (1.5%) positive CAPB patch test results in 2036 patients tested, with hairdressing products being the culprit exposure [46]. Saripalli, Achen, and Belsito conducted a retrospective analysis of patch test data collected from 1995 to 2001 in the USA and stated that 17 of 898 (19.7%) hairdressers had a positive test reaction regarding CAPB [54]. Schnuch et al. performed a retrospective analysis of data on patch testing from 1996 to 2008 from Germany and found that of 83,864 patients tested, 1812 (2.2%) had a positive result regarding CAPB [55]. Boonchai et al. examined trends in contact allergies to cosmetic ingredients in Thailand from 1999 to 2008, based on a highly selected patient population, and found that of 1247 patients, 121 (9.7%) had a positive patch test reaction for CAPB [39]. However, the high prevalence found mainly relates to the specific patient selection employed and is difficult to compare to other results. Wang et al. reviewed patch test results from 2000 to 2008 from the USA and stated that 9 of 206 (4.4%) patients showed a positive result for CAPB [64]. Pratt et al. reviewed patch test data from the North American Contact Dermatitis Group from 2001 to 2002 from the USA and stated that 137 of 4887 (2.8%) patients showed a positive result for CAPB [52]. Warshaw et al. looked at patch test reactions associated with cosmetics from 2001 to 2004 from the USA and showed that of 6621 patients tested, 84 (1.3%) had a positive test reaction for CAPB, with cosmetics being identified as the culprit exposure [67]. Davis et al. reviewed patch test data from 2001 to 2005 from the USA and found that 49 of 1093 (4.5%) patients provided a positive result for CAPB [40]. Toholka et al. conducted a retrospective analysis of patch test data from 2001 to 2010 in Australia and showed that 292 of 4297 (6.8%) patients had a positive patch test result for CAPB [60]. Warshaw et al. looked at patch test reactions associated with hair care products from 2001 to 2016 from the USA and showed that of 38,775 patients tested, 250 (0.6%) had a positive test reaction for CAPB, with hair care products being identified as the culprit exposure [68]. Suuronen, Pesonen, and Aalto-Korte reviewed patch test records at the Finnish Institute of Occupational Health in Finland from 2002 to 2009 and found that 2 of 1092 (0.2%) patients had positive results regarding CAPB [58]. Warshaw et al. looked at patch test data from the North American Contact Dermatitis Group from the USA from 2003 to 2004 and saw positive reactions for CAPB in 94 of 5137 (1.8%) patients. Li looked at patch test data from 2005 to 2006 from China and found that 42 of 429 (9.8%) patients had a positive result for CAPB, with cosmetics being identified as the culprit exposure [49]. Tomar et al. displayed patch test results from 2005 from India, in which 2 of 50 (4.0%) patients showed a positive result for CAPB, with cosmetics being the main source of exposure [61]. Fransway et al. evaluated patch test data from the North American Contact Dermatitis Group in the USA from 2007 to 2008 and showed that of 5082 patients tested, 56 (1.1%) had a positive patch test result against CAPB [43]. Tam et al. disclosed patch test results from the Massachusetts General Hospital Contact Dermatitis Clinic, USA, from 2007 to 2016 and stated that 12 of 2316 (0.5%) patients had a positive result regarding CAPB [59]. Warshaw et al. analyzed patch test data from the North American Contact Dermatitis Group from 2009 to 2010 from the USA and found that 4 of 4304 (0.1%) patients showed a positive result for CAPB [66]. Sundquist, Yang, and Pasha conducted a retrospective review of data from 2010 to 2016 from Canada and found that 2 of 555 (0.4%) patients had a positive patch test result for CAPB [57]. Veverka et al. looked at patch test data from 2011 to 2015 from the USA and found that of 2573 patients, 58 (2.3%) showed a positive patch test result for CAPB [63]. DeKoven et al. described patch test data from the North American Contact Dermatitis Group in the USA from 2013 to 2014 and outlined that of 4859 patients, 77 (1.5%) showed a positive patch test result for CAPB, with cosmetics being identified as the main source of exposure [41]. Garg et al. looked at patch test data from India from 2013 to 2015 and found that of 58 patients tested, 1 (1.7%) showed a positive result for CAPB, with cosmetics being identified as the main exposure source [44]. DeKoven et al. presented patch test data from the North American Contact Dermatitis Group in the USA from 2015 to 2016, in which 89 of 5592 (1.6%) of the tested patients provided a positive patch test result regarding CAPB, with cosmetics being the culprit exposure [42]. Salverda et al. conducted a cosmetovigilance survey and identified shampoos, conditioners, and make-up removers as the most frequently reported cosmetic products for allergies against CAPB [53]. Figure 4 depicts the pooled CAPB contact allergy prevalence in patients other than hairdressers, or rather with non-specified occupation/exposure, which is 1.9%.

Case reports and case series regarding CAPB are summarized in Table A7. There are two case reports and one case series on CAPB sensitization in hairdressers. In 1992 in Germany, Korting et al. described two cases of allergic contact dermatitis of the hands to CAPB in hairdressers (both female, age 22 and 28), which could be traced back to shampoo [75]. In 1992, Taniguchi et al. presented a case of allergic contact dermatitis of the hands and forearms to CAPB in a 22-year-old male Japanese hairdresser, in whom shampoo was identified as the culprit exposure [81]. In 1998, Lin-Hui and Sun reported on a positive patch test reaction to CAPB in a 47-year-old female hairdresser with chronic hand eczema, working for 30 years in Taiwan, in whom shampoo and hair dye were identified as sources of exposure [76].

Four case reports and one case series describe CAPB sensitization solely in people with an unspecified occupation. In 1991, Ross and White reported on a case of eyelid dermatitis due to CAPB in an eye make-up remover in a 60-year-old woman from the UK [80]. In 1998, Brand and Delaney described a case of severe allergic scalp dermatitis to CAPB in hair shampoo in a 50-year-old woman in Australia [74]. In 2001, Mowad published a case of allergic contact dermatitis of the trunk related to CAPB in a shampoo in a 75-year-old man in the USA [79]. In 2001, McFadden et al. reported on a series of cases (six women and one man, with an age ranging from 26 to 69 years) of CAPB allergy in the UK, with eye make-up remover and liquid soap identified as culprit exposures [77]. In 2004, Moreau and Sasseville described a case of allergic face dermatitis to CAPB in a facial cream in a 39-year-old Canadian woman [78].

## 4. Discussion

Hairdressers are exposed to a considerable amount of substances used in hair cosmetics, which mostly evince a considerable irritant and/or allergic potential. This accounts for a variety of substances, such as detergents, used, e.g., in shampoos, film-forming substances, e.g., in hairspray, as well as hair-waving agents in perming solutions—the present six target substances presenting an indicative set of important, common ingredients. It must be assumed that hairdressers handle these products much more often than clients—simply because of their daily work [16]—so that risk assessment tailored to the regular home user is probably unlikely to reflect the much greater occupational exposure of a professional. This has to be regarded as problematic in the highly skin-strained occupational group of hairdressers. Due to the impaired epidermal barrier function and the proinflammatory skin milieu which an irritant HE entails, irritants and allergens penetrate the skin barrier more easily and as a result, allergic HE might be acquired more easily than without pre-existing irritant damage. As there is still no causative therapy available for allergic HE (i.e., in terms of a type IV hypersensitivity, also called delayed-type hypersensitivity), allergen avoidance is the only feasible option. If this is not possible at the workplace, hairdressers might be subjected to precarious working situations, such as the necessity of changing profession or, in the worst case, withdrawal from the workforce. This highlights the serious consequences of HE in hairdressers, which can only be tackled by preventative measures if knowledge is collected about occupational hazards (e.g., ingredients in hair cosmetic products).

As a limitation of this review, the insufficient data situation regarding some of the investigated substances should be mentioned. These data gaps point to the necessity of more research on exposure and exposure-related contact dermatitis that needs to be conducted in the future to enable adequate risk assessment.

For cysteamine HCl, it could be shown that perm solutions and hair dyes are mainly identified as culprit exposures [73,85,86]. It must be assumed that hairdressers have a higher risk of acquiring quantum sensitization against cysteamine HCl compared to a consumer due to their occupational obligations. A current review on the differences between hairdressers and consumers in skin exposure to hair cosmetic products has shown that regarding coloring hair with permanent/oxidative hair color, hairdressers are 32 to 78 times higher exposed than consumers [16]. In the aforementioned review, Symanzik et al. further stress that information on consumer exposure on perming the hair is scarce, and self-use is highly improbable. Contrary to customers, hairdressers are subjected to various types of perming lotions (namely acid, alkaline, and exothermic types) and apply these two (for acid perms) to three (for alkaline perms) times a day, with a mean duration of 5.0 min per application (for acid and alkaline perms) conducted by 44.3 (for alkaline perms) and 97.5% (for acid perms) of hairdressers; 29.2% (alkaline perm) and 34.7% (acid perm) wear gloves whilst applying these lotions [16]. It is safe to assume that there is no home use of perming solutions, which is solely due to the complex winding technique with special rollers used when perming the hair. Home-user exposure thus is to be excluded for perming solutions and their ingredients.

It is not surprising that no data regarding the skin toxicity of PVP were found since it is know that PVP and eicosene alone are regarded as non-sensitizing; their copolymer, however, may induce skin sensitization [92]. Culprit exposures for PVP copolymers seem to be mainly skin care products such as moisturizers and lip products such as lipstick [88,89,90,91]. This suggests that hairdressers, who are also frequent consumers of the aforementioned products, may be exposed primarily through the well-groomed appearance expected in the hairdressing trade, rather than in the performance of their professional duties. Regarding consumers, PVP copolymers should also be kept in mind regarding sunscreens, as such cases have been reported previously [93,94].

Concerning the detergent SLES, it was to be expected that no data are available in respect of allergic potential [95], as regarding the closely related detergent sodium lauryl sulfate (SLS). In terms of irritant potential, SLES can, in contrast to other detergents such as SLS, be described as mild [96,97]. It is reasonable to presume that contemporary skin cleansers where SLES is used rather than stronger detergents, such as SLS, have improved in terms of skin barrier damage compared to the previous formulations available [98].

The results regarding cocamide DEA, which is widely used in shampoos and liquid soaps, lead to the assumption that this allergen does not seem to be of high relevance within the general population. Data from potential high-risk collectives in terms of exposure, such as hairdressers, however, are missing. The risk for this occupational group can thus not be conclusively estimated. From the results of the present review, culprit exposure to cocamide DEA mainly comes from cleansing products such as shampoo or topicals such as creams [82,83,84]. Given that hairdressers may be exposed to cocamide DEA when washing their clients’ hair with shampoo, the irritant potential of this surfactant should not be ignored [99].

CAPB is an amphoteric surfactant, frequently used in personal care products [100]. Allergic reactions to CAPB often present as eyelid, facial, scalp, and/or neck dermatitis, which can be traced back to the location in which exposure to a personal cleansing product is given [101]. In a paper published in 1996, Angelini et al. concluded that pure CAPB is not the allergen in patients with positive reactions to commercial CAPB [102]. This statement focuses on the extensively described problem of impurities in CAPB, which have caused allergic reactions rather than the substance itself. Industry stakeholders oftentimes refer back to the argument that purified grades of CAPB are unlikely to trigger allergic reactions, which is affirmed by a case series from 2007 by McFadden et al. [77]. It should be questioned whether non-purified grades of CAPB could be cheaper to purchase and would thus probably be used more often. The current cosmetic regulation regarding CAPB gives manufacturers of cosmetics plenty of rope for this question; there is no regulation on a mandatory use of purified grades of CAPB in cosmetics [103]. It should accordingly not be assumed that only purified grades of CAPB are used. With our meta-analysis, we could also show that hairdressers seem to have a 1.7-fold increased risk of developing a contact allergy to CAPB compared to controls who are not hairdressers (Figure 2).

At this juncture, it should be noted that the location of exposure should be considered, as hairdressers will likely have skin contact with hair dyes and perm solutions on their hands and also more often than a consumer. The degradation of hairdressers’ epidermal barrier function on the hands due to skin strain in everyday working life, associated with the onset of a proinflammatory milieu, raises the likelihood of developing occupational contact dermatitis to chemicals [8], not only to extreme allergens such as *p*-phenylenediamine, but also to low but repeated doses of, e.g., preservatives or fragrances [22], or weaker allergens such as those examined here. Although gloves should be worn when conducting hair coloring and perming services, previous research showed that the share of hairdressers actually wearing gloves is disillusioning. Indeed, hairdressers repeatedly reuse previously worn gloves [104] and contamination occurs as a result of improper use or when gloves are removed [105]. The breakthrough times of gloves used by hairdressers [106] are usually <10 min, and the fact that a share of the substances used in hairdressing products (e.g., *p*-phenylenediamine (PPD) used in hair colour) often penetrate glove material [107] results in the use of gloves not being as effective as intended [16].

From a methodological point of view, the presented case reports highlight the need for patch testing patients’ own products, e.g., moisturizers, lipsticks, sunscreens, perming solutions, etc., to identify causative allergens. In those case reports, patients’ own products were patch tested initially, then followed by further patch testing of the substances contained in the product [77,80,81,82,85,88,89,90,91,93,94]. This consecutive approach is indispensable to reliably identify a causal allergen, particularly in cases where commercial test allergens are not available. Thus, only by identifying causative allergens will it be possible to effectively avoid allergens and prevent the onset of allergic contact dermatitis. It should be mentioned, however, that for such patch testing of patient-specific products, specific knowledge in terms of the suitable preparation (test vehicle, test concentration, etc.) of these substances is necessary in order to obtain meaningful results and simultaneously minimize the risk associated with iatrogenic sensitization for the patient [108].

## 5. Conclusions

The findings of this study foremost show a lack of evidence published in the last 30 years relating to exposure to, and skin adverse effects from, the indicative set of six important detergent, film-forming, and hair restructuring agents studied. Only with regard to the more broadly patch-tested surfactant CAPB could a significantly increased risk of contact allergy in hairdressers be identified. This suggests, in line with results from other hairdressing cosmetic chemicals [109], that an estimated frequency of use by consumers is insufficient to determine hairdressing exposure. Thus, current standards of risk assessment do not effectively address the occupational risks associated with the use of hair cosmetics by hairdressers. The significant irritant and/or allergenic potential of substances used in hair cosmetics should prompt a reassessment of current risk assessment practices.

## Figures and Tables

**Table 1:** Eligibility criteria following the PECOS scheme.

Criterion	Inclusion	Exclusion
**P**articipants	Hairdressers, Patients, Products	None
**E**xposure	Exposure to (an) eligible chemical(s) †	n/a
**C**omparator	Clients, Consumers, normal Population (no or less exposure)	n/a
**O**utcome	Skin toxicity event (contact allergy, irritancy)	n/a
**S**tudy design	Experimental studies	Other qualitative studies
	Observational studies	
	Case reports	
	Case series	

† cysteamine hydrochloride (cysteamine HCl), usually used in perming solutions and also in hair dyes, polyvinylpyrrolidone (PVP) and polyvinylpyrrolidone (PVP) copolymers, which are used as film-forming components in, e.g., hairspray, as well as sodium laureth sulfate (SLES), cocamide diethanolamine (cocamide DEA), and cocamidopropyl betaine (CAPB), which are used as detergents in cosmetic applications.

**Table 2:** Summary of patch test results of original articles regarding cocamide diethanolamine (cocamide DEA).

Study	Study Period	Country	Positive Results for Cocamide DEA
DeKoven et al. [41]	2013–2014	USA	1 of 4859 (0.02%)
Toholka et al. [60]	2001–2010	Australia	1 of 4297 (0.02%)
Warshaw et al. [67]	2001–2004	USA	1 of 4304 (0.02%)
DeKoven et al. [42]	2015–2016	USA	2 of 5594 (0.04%)
Warshaw et al. [72]	2011–2012	USA	4 of 64,230 (0.1%)
Veverka et al. [63]	2011–2015	USA	2 of 2573 (0.1%)
Fransway et al. [43]	2007–2008	USA	4 of 5082 (0.1%)
Davis et al. [40]	2001–2005	USA	1 of 410 (0.2%)
Sundquist, Yang, and Pasha [57]	2010–2016	Canada	3 of 385 (0.8%)
Mertens, Gilissen, and Goossens [71]	1990–2015	Belgium	18 of 1767 (1.0%)
Aalto-Korte et al. [69]	1993–2011	Finland	25 of 2572 (1.0%)
Warshaw et al. [65]	2003–2004	USA	56 of 5137 (1.1%)
Warshaw et al. [66]	2009–2010	USA	28 of 609 (4.6%)

Cocamide DEA, cocamide diethanolamine; USA, United States of America.

## Data Availability

Data, code, and other materials other than those included in the PROSPERO registration [20] and the previously published protocol [15] are available from the corresponding author upon reasonable request.

## Appendix A

Search string for Cysteamine hydrochloride (Cysteamine HCl): ((Cysteamine hydrochloride) OR (156-57-0) OR (2-Aminoethanethiol hydrochloride) OR (Cysteamine HCl) OR (2-Mercaptoethylamine hydrochloride) OR (Cysteaminium chloride) OR (Mercaptamine hydrochloride) OR (ETHANETHIOL, 2-AMINO-, HYDROCHLORIDE) OR (2-Thioethylamine hydrochloride) OR (Mercaptoethylamine hydrochloride) OR (2-aminoethane-1-thiol hydrochloride) OR (2-Mercaptoethylammonium chloride) OR (beta-Mercaptoethylamine hydrochloride) OR (2-aminoethanethiol;hydrochloride) OR (2-Mercaptoethylamine HCl) OR (Cysteamine chlorohydrate) OR (CI 9148) OR (Ethylamine, 2-mercapto-, hydrochloride) OR (thioethanolamine hydrochloride) OR (cysteamine-hcl) OR (beta-Mercaptoaethylamin chlorhydrat) OR (Cystaran (TN)) OR ((beta)-MEA Hydrochloride) OR (2-aminoethanthiol hydrochloride) OR (2-amino-ethanethiol hydrochloride) OR (2-mercaptoethanamine hydrochloride)) AND (("1991/01/01"[Date - Publication] : "3000"[Date - Publication])) AND (Allergens[MeSH] OR Haptens[MeSH] OR agents, contact sensitizing[MeSH] OR allergic OR Dermatitis, Allergic Contact[MeSH] OR Dermatitis, Contact[MeSH] OR contact allergy OR Skin Tests[MeSH] OR Local Lymph Node Assay[MeSH] OR guinea pig maximization test OR Patch Tests[MeSH] OR Skin Irritancy Tests[MeSH] OR contact dermatitis OR contact urticaria OR contact sensitization OR Occupational Diseases[MeSH] OR work related)

Search string for Polyvinylpyrrolidone (PVP): ((Polyvinylpyrrolidone (PVP)) OR (9003-39-8) OR (1-vinyl-2-pyrrolidone) OR (N- vinyl pyrrolidone) OR (N-vinyl-2-pyrrolidinone) OR (N-vinylpyrrolidone)) AND (("1991/01/01"[Date - Publication] : "3000"[Date - Publication])) AND (Allergens[MeSH] OR Haptens[MeSH] OR agents, contact sensitizing[MeSH] OR allergic OR Dermatitis, Allergic Contact[MeSH] OR Dermatitis, Contact[MeSH] OR contact allergy OR Skin Tests[MeSH] OR Local Lymph Node Assay[MeSH] OR guinea pig maximization test OR Patch Tests[MeSH] OR Skin Irritancy Tests[MeSH] OR contact dermatitis OR contact urticaria OR contact sensitization OR Occupational Diseases[MeSH] OR work related)

Search string for Polyvinylpyrrolidone (PVP) copolymers: ((PVP/acrylates/lauryl methacrylate copolymer) OR (PVP/decene copolymer) OR (PVP/dimethylaminoethylmethacrylate copolymer) OR (PVP/DMAPA acrylates copolymer) OR (PVP/eicosene copolymer) OR (28211-18-9) OR (PVP/hexadecene copolymer) OR (PVP/VA copolymer) OR (25086-89-9) OR (PVP/VA/itaconic acid copolymer) OR (PVP/VA/vinyl propionate copolymer) OR (PVP/vinyl caprolactam/DMAPA acrylates copolymer)) AND (("1991/01/01"[Date - Publication] : "3000"[Date - Publication])) AND (Allergens[MeSH] OR Haptens[MeSH] OR agents, contact sensitizing[MeSH] OR allergic OR Dermatitis, Allergic Contact[MeSH] OR Dermatitis, Contact[MeSH] OR contact allergy OR Skin Tests[MeSH] OR Local Lymph Node Assay[MeSH] OR guinea pig maximization test OR Patch Tests[MeSH] OR Skin Irritancy Tests[MeSH] OR contact dermatitis OR contact urticaria OR contact sensitization OR Occupational Diseases[MeSH] OR work related)

Search string for Sodium laureth sulfate (SLES): ((Sodium laureth sulfate) OR (9004-82-4)) AND (("1991/01/01"[Date - Publication] : "3000"[Date - Publication])) AND (Allergens[MeSH] OR Haptens[MeSH] OR agents, contact sensitizing[MeSH] OR allergic OR Dermatitis, Allergic Contact[MeSH] OR Dermatitis, Contact[MeSH] OR contact allergy OR Skin Tests[MeSH] OR Local Lymph Node Assay[MeSH] OR guinea pig maximization test OR Patch Tests[MeSH] OR Skin Irritancy Tests[MeSH] OR contact dermatitis OR contact urticaria OR contact sensitization OR Occupational Diseases[MeSH] OR work related)

Search string for Cocamide diethanolamine (Cocamide DEA): ((68603-42-9) OR (Cocamide diethanolamine) OR (Cocamide DEA) OR (N,N-bis(2-hydroxyethyl) coco fatty acid diethanolamide) OR (coconut fatty acid diethanolamide, cocoyl diethanolamide) OR (coconut oil acid diethanolamide)) AND (("1991/01/01"[Date - Publication] : "3000"[Date - Publication])) AND (Allergens[MeSH] OR Haptens[MeSH] OR agents, contact sensitizing[MeSH] OR allergic OR Dermatitis, Allergic Contact[MeSH] OR Dermatitis, Contact[MeSH] OR contact allergy OR Skin Tests[MeSH] OR Local Lymph Node Assay[MeSH] OR guinea pig maximization test OR Patch Tests[MeSH] OR Skin Irritancy Tests[MeSH] OR contact dermatitis OR contact urticaria OR contact sensitization OR Occupational Diseases[MeSH] OR work related)

Search string for Cocamidopropyl betaine (CAPB): ((Cocamidopropyl betaine) OR (61789-40-0) OR (cocamidopropylbetaine)) AND (("1991/01/01"[Date - Publication] : "3000"[Date - Publication])) AND (Allergens[MeSH] OR Haptens[MeSH] OR agents, contact sensitizing[MeSH] OR allergic OR Dermatitis, Allergic Contact[MeSH] OR Dermatitis, Contact[MeSH] OR contact allergy OR Skin Tests[MeSH] OR Local Lymph Node Assay[MeSH] OR guinea pig maximization test OR Patch Tests[MeSH] OR Skin Irritancy Tests[MeSH] OR contact dermatitis OR contact urticaria OR contact sensitization OR Occupational Diseases[MeSH] OR work related)

## Appendix B

Appendix B comprises all the publication record forms (PRFs) for the included original articles as well as case series and/or case reports.

**Table A1:** Publication record forms (PRFs) of original articles for cysteamine hydrochloride (cysteamine HCl).

Study	Year (Range)	Country	Study Design	Patch Testing	Patch Testing Context	Population Tested	Female (%)	Age (Years)	No. of Hairdresser Tested	No. of Hairdressers with a Positive Result	No. of Others Tested	No. of Others Tested with a Positive Result
Ito et al. [73]	2012–2014	Japan	epidemiological sample	yes	patch test special series	all patch-tested patients	87	58 (mean)	n/a	n/a	192	26
Schwensen et al. [56]	2002–2011	Denmark	epidemiological sample	yes	patch test special series	hairdressers	83	30.8 (mean)	12	1	n/a	n/a

n/a, not applicable.

**Table A2:** Publication record forms (PRFs) of case series and case reports for cysteamine hydrochloride (cysteamine HCl).

Study	Year (Range)	Country	Sex	Age	Occupation	Working Years	Own Products Tested
Isaakson and van der Walle [85]	2007	Sweden	1 female	53	hairdresser	27	permanent-wave solution
Landers, Law, and Storrs [86]	2002	USA	1 female	38	hairdresser	n/a	permanent-wave solution
Nishioka, Koizumi, and Takita [87]	2012–2017	Japan	4 females 3 males	22 to 73	hairdresser	n/a	n/a

n/a, not applicable; USA, United States of America.

**Table A3:** Publication record forms (PRFs) of case reports for polyvinylpyrrolidone (PVP) copolymers.

Study	Year (Range)	Country	Sex	Age	Occupation	Working Years	Own Product Tested
Buonomo and Warshaw [88]	2021	USA	1 female	25	n/a	n/a	moisturizer
Pastor et al. [89]	2008	Spain	1 female	20	n/a	n/a	lipstick
Quartier et al. [90]	2006	Belgium	1 female	28	n/a	n/a	lipstick
Scheman and Cummins [91]	1998	USA	1 female	53	n/a	n/a	skin care products

n/a, not applicable; USA, United States of America.

**Table A4:** Publication record forms (PRFs) of original articles for cocamide diethanolamine (cocamide DEA).

Study	Year (Range)	Country	Study Design	Patch Testing	Patch Testing Context	Population Tested	Female (%)	Age (years)	No. of Hairdresser Tested	No. of Hairdressers with a Positive Result	No. of Others Tested	No. of Others Tested with a Positive Result
Mertens, Gilissen, and Goossens [71]	1990–2015	Belgium	monocentric retrospective study	yes	epidemiological sample	all patch-tested patients	n/a	n/a	n/a	6	1767	18
Aalto-Korte et al. [69]	1993–2011	Finland	monocentric retrospective study	yes	epidemiological sample	occupational patch-tested patients	n/a	n/a	n/a	n/a	2572	25
Warshaw et al. [67]	2001–2004	USA	multicentric retrospective study	yes	consecutive patient	all patch-tested patients	66	n/a	n/a	n/a	609	28
Davis et al. [40]	2001–2005	USA	monocentric retrospective study	yes	consecutive patients	all patch-tested patients	66.8	55.1 (mean)	n/a	n/a	410	1
Toholka et al. [60]	2001–2010	Australia	monocentric retrospective study	yes	consecutive patients	all patch-tested patients	65	40 (mean)	n/a	n/a	4297	1
Warshaw et al. [65]	2003–2004	USA	multicentric retrospective study	yes	consecutive patients	all patch-tested patients	65.2	47.5 (mean)	n/a	n/a	5137	56
Fransway et al. [43]	2007–2008	USA	multicentric retrospective study	yes	consecutive patients	all patch-tested patients	64.3	n/a	n/a	n/a	5082	4
Warshaw et al. [66]	2009–2010	USA	multicentric retrospective study	yes	consecutive patients	all patch-tested patients	77.9	48.4 (mean)	n/a	n/a	4304	1
Sundquist, Yang, and Pasha [57]	2010–2016	Canada	monocentric retrospective study	yes	consecutive patients	all patch-tested patients	71.1	4 to 92	n/a	n/a	385	3
Warshaw et al. [72]	2011–2012	USA	multicentric retrospective study	yes	consecutive patients	all patch-tested patients	68.6	50 (mean)	n/a	n/a	4230	4
Veverka et al. [63]	2011–2015	USA	monocentric retrospective study	yes	consecutive patients	all patch-tested patients	67.4	53.4 (mean)	n/a	n/a	2573	2
DeKoven et al. [41]	2013–2014	USA	multicentric retrospective study	yes	consecutive patients	all patch-tested patients	70	50 (mean)	n/a	n/a	4859	1
DeKoven et al. [42]	2015–2016	USA	multicentric retrospective study	yes	consecutive patients	all patch-tested patients	72	50 (mean)	n/a	n/a	5594	2
Grey et al. [70]	2015–2016	USA	others	yes	patch test special series	all patch-tested patients	78.7	55.2 (mean)	n/a	n/a	47	4

n/a, not applicable, USA, United States of America.

**Table A5:** Publication record forms (PRFs) of case series and case reports for cocamide diethanolamine (cocamide DEA).

Study	Year (Range)	Country	Sex	Age	Occupation	Working Years	Own Product Tested
Fowler [84]	1997	USA	1 female 2 males	40 47, 28	n/a manufacturing, mechanics	n/a	personal care products
Dejobert et al. [83]	2005	France	1 female	27	n/a	n/a	shampoo
Badaoui et al. [82]	2012–2014	France	4 females 2 males	51.6 (mean)	n/a	n/a	mycoster cream, cyteal solution

n/a, not applicable; USA, United States of America.

**Table A6:** Publication record forms (PRFs) of original articles for cocamidopropyl betaine (CAPB).

Study	Year (Range)	Country	Study Design	Patch Testing	Patch Testing Context	Population Tested	Female (%)	Age (years)	No. of Hairdresser Tested	No. of Hairdressers with a Positive Result	No. of Others Tested	No. of Others Tested with a Positive Result
De Groot, van der Walle, and Weyland [26]	1991–1994	Netherlands	multicentric retrospective study	yes	consecutive patients	all patch-tested patients	n/a	n/a	217	8	564	9
Armstrong et al. [25]	1991–1998	UK	monocentric retrospective study	yes	consecutive patients	all patch-tested patients	n/a	n/a	184	1	10,614	28
Uter et al. [24]	1995–2002	Austria, Germany, Switzerland	multicentric retrospective study	yes	epidemiological sample	hairdressers	100	24 (mean)	884	27	1217	24
Uter et al. [23]	2003–2006	Germany	multicentric retrospective study	yes	epidemiological sample	hairdressers	100	26 (mean)	432	14	614	17
Uter et al. [22]	2007–2012	Austria, Germany, Switzerland	multicentric retrospective study	yes	epidemiological sample	hairdressers	100	24 (mean)	744	22	1903	37
Van der Walle and Brunsveld [62]	1989–1992	Netherlands	monocentric retrospective study	yes	epidemiological sample	hairdressers	92.2	16 to 52	103	4	n/a	n/a
Lyons et al. [50]	1993–2010	Australia	monocentric retrospective study	yes	epidemiological sample	hairdressers	96	23 (mean)	164	1	n/a	n/a
Schwensen et al. [56]	2002–2011	Denmark	epidemiological sample	yes	epidemiological sample	hairdressers	n/a	16 to 79	287	1	1995	n/a
Krecisz, Kiec-Swierczynska, and Chomiczewska [48]	2011	Poland	epidemiological sample	yes	patch test special series	hairdressers	96	18 (mean)	139	1	n/a	n/a
Carøe, Ebbehøj, and Agner [6]	2006–2011	Denmark	epidemiological sample	yes	epidemiological sample	hairdressers	99.7	25 (mean)	381	18	n/a	n/a
Hillen, Grabbe, and Uter [47]	1993–2003	Germany	multicentric retrospective study	yes	epidemiological sample	all patch-tested patients	n/a	n/a	n/a	n/a	1021	48
Patel and Belsito [51]	1995–2005 2005–2010	USA	multicentric retrospective study	yes	consecutive patients	all patch-tested patients	n/a	n/a	n/a	n/a	1831	35
Hasan et al. [46]	1995–1997	Finland	multicentric retrospective study	yes	patch test special series	all patch-tested patients	n/a	n/a	n/a	n/a	2036	30
Saripalli, Achen, and Belsito [54]	1995–2001	USA	multicentric retrospective study	yes	consecutive patients	all patch-tested patients	n/a	n/a	n/a	n/a	898	17
Schnuch et al. [55]	1996–2009	Germany	multicentric retrospective study	yes	consecutive patients	all patch-tested patients	n/a	n/a	n/a	n/a	83,864	1.812
Boonchai, Desomchoke, and Iamtharachai [39]	1999–2008	Thailand	monocentric retrospective study	yes	epidemiological sample	all patch-tested patients	81	8 to 84	n/a	n/a	1247	121
Wang et al. [64]	2000–2008	USA	multicentric retrospective study	yes	patch test special series	all patch-tested patients	94.8	53.8 (mean)	n/a	n/a	206	9
Pratt et al. [52]	2001–2002	USA	Multicentric retrospective study	yes	consecutive patients	all patch-tested patients	n/a	n/a	n/a	n/a	4887	137
Warshaw et al. [67]	2001–2004	USA	multicentric retrospective study	yes	consecutive patients	all patch-tested patients	66	49.3 (mean)	n/a	n/a	6621	84
Davis et al. [40]	2001–2005	USA	multicentric retrospective study	yes	consecutive patients	all patch-tested patients	66.8	55.1 (mean)	n/a	n/a	1093	49
Toholka et al. [60]	2001–2010	Australia	monocentric retrospective study	yes	consecutive patients	all patch-tested patients	65	41 (mean)	n/a	n/a	4297	292
Warshaw et al. [68]	2001–2016	USA	multicentric retrospective study	yes	consecutive patients	all patch-tested patients	n/a	n/a	n/a	n/a	38,775	250
Suuronen, Pesonen, and Aalto-Korte [58]	2002–2009	Finland	monocentric retrospective study	yes	epidemiological sample	all patch-tested patients	n/a	n/a	n/a	n/a	1092	2
Warshaw et al. [65]	2003–2004	USA	multicentric retrospective study	yes	consecutive patients	all patch-tested patients	65.2	n/a	n/a	n/a	5137	94
Li [49]	2005–2006	China	monocentric retrospective study	yes	patch test special series	all patch-tested patients	75	9 to 81	n/a	n/a	429	42
Tomar et al. [61]	2005	India	monocentric retrospective study	yes	epidemiological sample	all patch-tested patients	70	16 to 55	n/a	n/a	50	2
Fransway et al. [43]	2007–2008	USA	multicentric retrospective study	yes	consecutive patients	all patch-tested patients	n/a	n/a	n/a	n/a	5082	56
Tam et al. [59]	2007–2016	USA	monocentric retrospective study	yes	consecutive patients	all patch-tested patients	73.4	47.7 (mean)	n/a	n/a	2316	12
Warshaw et al. [66]	2009–2010	USA	multicentric retrospective study	yes	consecutive patients	all patch-tested patients	67.9	48.4 (mean)	n/a	n/a	4304	4
Salverda et al. CAPB [53]	2009–2011	Netherlands	other (cosmetovigilance)	yes	consecutive patients	n/a	n/a	41 (mean)	n/a	n/a	n/a	n/a
Sundquist, Yang, and Pasha [57]	2010–2016	Canada	monocentric retrospective study	yes	consecutive patients	all patch-tested patients	71.1	4 to 92	n/a	n/a	555	2
Gregoriou et al. [45]	2010–2019	Greece	monocentric retrospective study	yes	patch test special series	all patch-tested patients	89.5	13 to 87	136	20	226	11
Veverka et al. [63]	2011–2015	USA	monocentric retrospective study	yes	consecutive patients	all patch-tested patients	67.4	53.4 (mean)	n/a	n/a	2573	58
DeKoven et al. exposure [41]	2013–2014	USA	multicentric retrospective study	yes	consecutive patients	all patch-tested patients	n/a	n/a	n/a	n/a	4859	77
Garg et al. [44]	2013–2015	India	monocentric retrospective study	yes	consecutive patients	all patch-tested patients	86.2	36.3 (mean)	n/a	n/a	58	1
DeKoven et al. [42]	2015–2016	USA	multicentric retrospective study	yes	consecutive patients	all patch-tested patients	n/a	n/a	n/a	n/a	5592	89

n/a, not applicable; UK, United Kingdom; USA, United States of America.

**Table A7:** Publication record forms (PRFs) of case series and case reports for cocamidopropyl betaine (CAPB).

Study	Year (Range)	Country	Sex	Age	Occupation	Working Years	Own Product Tested
Korting et al. [75]	1992	Germany	2 females	n/a	hairdresser	n/a	shampoos
Taniguchi et al. [81]	1992	Japan	male	22	hairdresser	n/a	shampoos
Lin-Hui and Sun [76]	1998	Taiwan	female	47	hairdresser	30	shampoo, hair dye
Ross and White [80]	1991	UK	female	60	n/a	n/a	eye make-up remover
Brand and Delaney [74]	1998	Australia	female	50	n/a	n/a	shampoos
Mowad [79]	2001	USA	male	75	n/a	n/a	shampoo
McFadden [77]	2001	UK	6 females 1 male	26–69	n/a	n/a	eye make-up remover, liquid soap
Moreau and Sasseville [78]	2004	Canada	female	39	n/a	n/a	facial creams

n/a, not applicable; UK, United Kingdom; USA, United States of America.
